# From inside to outside: exploring extracellular antimicrobial histone-derived peptides as multi-talented molecules

**DOI:** 10.1038/s41429-024-00744-0

**Published:** 2024-06-13

**Authors:** Carolina Muñoz-Camargo, Juan C. Cruz

**Affiliations:** Grupo de investigación en Nanobiomateriales, Ingeniería Celular y Bioimpresión (GINIB), Departamento de Ingeniería Biomédica, Universidad de los Andes, Bogotá, Colombia

**Keywords:** Drug discovery and development, Antibiotics

## Abstract

The emergence of bacterial resistance to antibiotics poses a global health threat, necessitating innovative solutions. The contemporary challenge lies in bacterial resistance, impacting morbidity, mortality, and global economies. Antimicrobial peptides (AMPs) offer a promising avenue for addressing antibiotic resistance. The Antimicrobial Peptide Database catalogs 3569 peptides from various organisms, representing a rich resource for drug development. Histones, traditionally recognized for their role in nucleosome structures, have gained attention for their extracellular functions, including antimicrobial and immunomodulatory properties. This review aims to thoroughly investigate antimicrobial peptides derived from histones in various organisms, elucidating their mechanisms. In addition, it gives us clues about how extracellular histones might be used in drug delivery systems to fight bacterial infections. This comprehensive analysis emphasizes the importance of histone-derived peptides in developing innovative therapeutic strategies for evolving bacterial challenges.

## Introduction

Since the origin of bacteria approximately 3.5 billion years ago, they have co-evolved with natural antibiotics present in their environment. Contrary to the common belief that exposure to these antibiotics is confined to the antibiotic era, there is evidence suggesting that this type of molecules were a natural source before the antibiotic era. For example, traces of tetracycline have been found in human skeletons dating back to 350–550 BC (before the Common Era) [1] due to tetracycline binds to bone tissue through chelation, forming complexes with calcium ions in the hydroxyapatite matrix of bones and teeth. The possible explanation for this discovery is that these ancient people’s activities and diet included exposure to naturally occurring molecules of tetracycline (soil microorganism e.g *Streptomyces*). Therefore, tracing other antibiotics in ancient populations is considerably more challenging, primarily because of the difficulty in detecting them.

When the antibiotic era began in 1940, Alexander Fleming, who had discovered penicillin two decades before, cautioned against the possibility of penicillin resistance developing if the drug was administered sparingly and for brief periods. Penicillin is the most common example of an antibiotic, defined as a drug capable of killing or halting bacterial growth [2]. The period between 1950 and 1970 was the golden age of discovering new antibiotics, but since then, this area of research has focused on modifying existing classes of antibiotics to combat infections caused by emerging and re-emerging pathogens. Currently, the problem of bacterial resistance and multi-resistance to available antibiotics is global. Data for the United States indicate that every year, 2.8 million people are infected with antibiotic-resistant bacteria, and 35,000 die directly from these infections, according to the CDC’s 2019 Antibiotic Resistance (AR) Threats Report [3]. The Organization for Economic Cooperation and Development (OECD) estimated that between 2015 and 2050, antibiotic resistance may claim around 2.4 million lives in Europe, North America, and Australia [4]. Antibiotic resistance might impact 1.1% of gross domestic product (GDP) reduction and may exceed an estimated US$1 trillion annually after 2030 across the globe, in a low-impact scenario [5]. Consequently, treatment options for existing or multi-resistant infections are limited, resulting in increased rates of morbidity and mortality.

On the other hand, alternatives to conventional antibiotics, such as antimicrobial peptides (AMPs), have been studied for over 50 years. The Antimicrobial Peptide Database reports 3569 antimicrobial peptides from a variety of organisms, ranging from bacteria to mammals [6]. Some of those AMPs are often truncated versions of larger proteins within the cell. For example, histones are proteins known for their function in the cell nucleus; nevertheless, they can also be found in the cytoplasm and in extracellular space. The pieces that were made by proteolysis were named histone-derived peptides, and they were found to be able to fight both bacteria and inflammation. The discovery of new molecules with antimicrobial potential has been impacted using artificial intelligence algorithms, enhancing the strategy of protein or genome fragmentation in search of sequences with antimicrobial peptide characteristics in a fraction of the experimental time and with reduced in vitro selection costs [7].

**Fig. 1:**
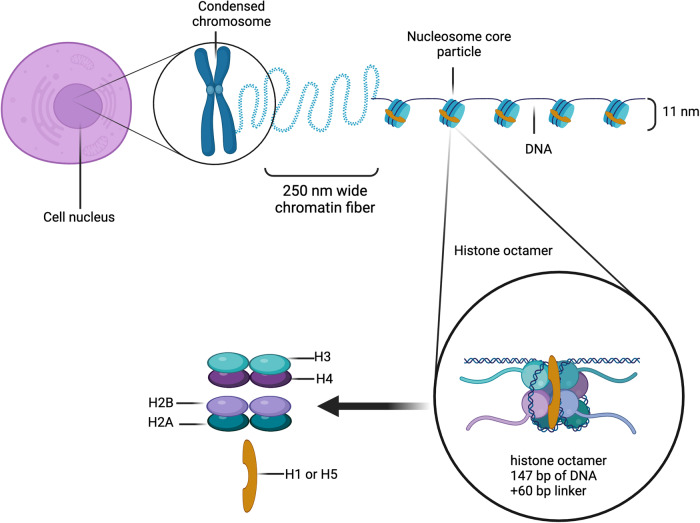
Packaging of DNA within eukaryotic cells. Nucleosomes and their conformation. DNA is packaged into octamers coupled with histones H2A, H2B, H3, and H4. Additionally, there is the presence of the linker histone H1 or H5, depending on the organism. Histone mRNAs undergo strict regulation and are abundant specifically during the S-phase, ensuring a sufficient supply of histone proteins essential for the packaging of recently duplicated DNA. Histones, highly conserved across evolution, constitute the basic building block of chromatin known as the nucleosome, responsible for enclosing the freshly replicated chromosomal DNA. “Created with BioRender.com”

Histones are better known as the major protein components of the nucleosome structures in eukaryotic cells. There are two main types of histones based on what they do: linker histones (Histone H1), which keep DNA loops closed and the nucleosome structures tight, and core histones (Histone H2A, H2B, H3, and H4), which put together an eight-part complex to make the nucleosome [8] (Fig. 1). The main functions of histones are always related to their interaction with DNA and their participation in the regulation of gene expression [9]. However, there is growing evidence that histones in extracellular fluid as well as on the surface of intact cells may be involved in a broad spectrum of biological functions, such as apoptosis [10], immunomodulatory properties, [11–14] action on injured tissue, [15, 16] and antibacterial activities [16].Researchers have found that one way cells respond to microorganisms is by storing histone-derived antimicrobial peptides (HDAPs) in the cytoplasm and on the plasma membrane, where they kill microbes.

Considering the above, the purpose of this review is to describe the research that has been done on antimicrobial peptides made from different organisms’ histones, how they work, what role they play in other immune biological functions, and the perspectives to use them in clinical settings.

## Histones and antimicrobial peptides: an intriguing association

AMPs are indispensable components of the innate immune system in various species, including humans, animals, and plants, and have become an interesting alternative to conventional antibiotics [17]. Thus, they are capable of being applied to treat various microorganisms, such as bacteria, fungi, viruses, and even drug-resistant ones. Similarly, HDAPs are reported to actively participate in the immune defenses of different organisms and are a special group of peptides that are produced by the proteolytic cleavage of unacetylated histones, and their principal characteristics are similar to those of other AMPs: low molecular weight, amphipathicity, and positive charge. (Table 1).

**Table 1:** Principal biological activities reported for HDAPs

Source	Protein/Peptide	Charge	Sequence	Uniprot ID	Biological activity (MIC)	Ref
H1-human terminal ileal mucosa	Full length H1 213 bp	+39.2	MSVELEEALPVTTAEGMAKKVTKAGGSAALSPSKKRKNSKKKNQPGKYSQLVVETIRRLGERNGSSLAKIYTEAKKVPWFDQQNGRTYLKYSIKALVQNDTLLQVKGTGANGSFKLNRKKLEGGGERRGAPAAATAPAPTAHKAKKAAPGAAGSRRADKKPARGQKPEQRSHKKGAGAKKDKGGKAKKTAAAGGKKVKKAAKPSVPKVPKGRK	*Q92522*	Antimicrobial activity *S. typhimurium* 6.95 μg/ml	[48]
H1 fragment-human terminal ileal mucosa	Fragment H1 10 bp	+2	KLNKKAASGE			
H1 fragment-human terminal ileal mucosa	Fragment H1 10 bp	+5	KAKSPKKAKA			
H1 fragment fish *Paralichthys olivaceus* *Olive flounder* *testes*	fH1LP 40 bp	+5	AEVPPAPAPAPAKAAKKKVVKPKKVGPSVREIIIEAVSAS	*I3QD85*	*Antimicrobial activity* *A. hydrophila* *B. subtilis 8.0* *µg/mL* *S. aureus 2.8* *µg/mL Streptococcus iniae 30* *µg/mL* *E. coli D31 1.4* *µg/mL* *V. parahaemolyticus 12* *µg/mL* *C. albicans 2* *µg/mL*	[39]
H1 fragment fish *Salmo salar* Atlantic salmon skin mucus	SAMP H1 30 bp	+6	AEVAPAPAAAAPAKAPKKKAAAKPKKAGPS	*P84408*	Antibacterial activity *E. coli* D31 12 µg/mL *A. salmonicida* 4 µg/mL *V. anguillarum* 1 µg/mL *S. enterica* 4 µg/mL *Bacillus subtilis* 2 µg/mL *Listeria ivanovii* 2 µg/mL	[38, 80]
H1 fragment *Oncorhynchus mykiss* Rainbow trout Fragment 138-154 Skin secretions	Oncorhyncin II 60 bp	+30	KAVAAKKSPKKAKKPATPKKAAKSPKKVKKPAAAAKKAAKSPKKATKAAKPKAAKPKAAKAKKAAPKKK	*P06350*	Antibacterial activity *M. luteus* 0.8 µM *P. citreus* 0.6 µM *E. coli* 0.8 µM *L. anguillarum* 0.8 µM	[81]
H1 fragment *Oncorhynchus mykiss* *Skin secretions*	Oncorhyncin III 69 bp	+14	PKRKSATKGDEPARRSARLSARPVPKPAAKPKKAAAPKKAVKGKKAAENGDAKAEAKVQAAGDGAGNAK	*P83287*	Antibacterial activity *A. viridans* 0.12 µM *B. subtilis* 0.5 µM *M. luteus* 0.25 µM *P. citreus* 0.12 µM *A. hydrophila* > 0.5 µM *A. salmonicida* > 0.5 µM *E. coli* 0.5 µM *L. anguillarum* 0.5 µM [79]	[82]
H2A human	Full length H2A 130 bp	+16.4	MSGRGKQGGKARAKAKSRSSRAGLQFP**VGRV**HRLLRKGNYAERVGAGAPVYMAAVLEYLTAEILELAGNAARDNKKTRIIPRHLQLAIRNDEELNKLLGKVTIAQGGVLPNIQAVLLPKKTESHHKAKGK	*Q6FI13*	Antibacterial Activity *E. coli* > 10 µg/mL *S. aureus* > 10 µg/mL	[2]
*H2A* *Litopenaeus vannamei* hemocytes	Full length H2A 126 bp	+17	MSGRGKGGKVKGKSKSRSSRAGLQFPVGRIHRLLRKGNYAERVGAGAPVYLAAVMEYLAAEVLELAGNAARDNKKTRIVPRHLQLAIRNDEELNKLLSGVTIAQGGVLPNIQAVLLPKKTEKK	*Q6PV61*	Antibacterial Activity *M. luteus* 4.5 µM	[3]
H2A fragment *Parasilurus asotus* (Catfish) Skin mucus	Parasin I 19 bp	+6	SGRGKQGGKARAKATRSS	*NR*	Antibacterial activity *A. salmonicida* 10 µg/mL *C. aquatilis* 5 µg/mL *Y. rukeri* 15 µg/mL *E. ictaluri* 10 µg/mL *L. garvieae* 10 µg/mL *E. coli* 1 µg/mL *S. typhimurium* 2 µg/mL *P. putida* 2 µg/mL *B. subtilis* 1 µg/mL *S. aureus* 2 µg/mL *S. mutans* 1 µg/mL	[16, 83]
*H2A fragment* *Bufo bufo gargarizans* Amphibian stomach	Buforin I 39 bp	+12	AGRGKQGGKVRAKAKTRSSRAGLQFPVGRVHRLLRKGNY	*P55897*	Antimicrobial activity *E. coli 4* µg/mL *P. putida* 4 µg/mL *S. typhimurium* 8 µg/mL *B. subtilis* 2 µg/mL *S. aureus* 4 µg/mL *S. mutans* 2 µg/mL *C. albicans* 4 µg/mL	[29]
*H2A fragment* *Bufo bufo gargarizans* *Amphibian stomach*	Buforin II 21 bp	+6	TRSSRAGLQFPVGRVHRLLRK	*P55897*	Antimicrobial activity *E. coli* 4 µg/mL *P. putida* 2 µg/mL *S. typhimurium* 2 µg/mL *B. subtilis* 4 µg/mL *S. aureus* 4 µg/mL *S. mutans* 8 µg/mL *C. albicans* 1 µg/mL Endotoxin activity Binding LPS and LTA [40, 41, 43, 53, 71]	[25, 30, 32, 84, 85]
*H2A fragment* *Bufo bufo gargarizans* *Amphibian stomach*	Buforin IIb 21 bp	+7	RAGLQFPVGRLLRRLLRRLLR	*P55897*	Antimicrobial activity *E. coli* 1 µg/mL *P. putida* 2 µg/mL *S. typhimurium* 1 µg/mL *B. subtilis* 1 µg/mL *S. aureus* 1 µg/mL *S. mutans* 0.5 µg/mL *C. albicans* 2 µg/mL	[21]
H2A Fragment *Designed*	DesHDAP1 20 bp	+5.25	ARDNKKTRIWPRHLQLAVRN	*NR*	*Antimicrobial activity* *E. coli* 9.4 µM *S. marcescens* 3.7 µM *B. subtilis* 8.3 µM *S. aureus* 4.0 µM *E. faecalis* 4.0 µM	[34, 35]
*H2A fragment* *Cynoglossus semifasciatus* *Fish Blood*	Teleostin 52 bp	+12.25	MSGRGKTGGKARAKAKTRSSRAGLQFPVGRVHRLLRKGNYAERVGAGAPVYL	*G0YP06*	Apparent antibacterial activity	[52]
*H2A fragment* *Tachysurus jella* *Fish blood*	Teleostin 52 bp	+12.25	MSGRGKTGGKARAKAKTRSSRAGLQFPVGRVHRLLRKGNYAERVGAGAPVYL	*G0YP07*	Apparent antibacterial activity	[52]
*H2A fragment* *Hippoglossus hippoglossus* *Skin mucus*	Hipposin 51 bp	+15	SGRGKTGGKARAKAKTRSSRAGLQFPVGRVHRLLRKGNYAHRVGAGAPVYL	*P59890*	Antibacterial activity *B. subtilis* 1.3 µM L. corenyformis 0.6 µM *L. ivanovii* 0.6 µM S. epidermis 10 µM *E. coli* 2.5 µM	[86, 87]
*H2A fragment* *Haliotis discus discus* *Digestive gland*	Abhisin 40 bp	+12.25	MSGRGKGGKTKAKAKSRSSRAGLQFPVGRIHRLLRKGNYA	*NR*	Antibacterial activity *L.monocytogenes 250* *μg/mL* *V. ichthyoenteri 250* *μg/mL*	[55]
*Fragment H2A* *Himantura pastinacoides* *Blood*	Himanturin 39 bp	+8.25	KAKSRSSRAGLQFPVGRVHRLLRKGNYAERVGAGAPVYL	*NR*	Antimicrobial agent	[4]
Fragment H2A Neoharriotta pinnata Blood	Harriotin 80 bp	+12.25	MSGRGKTGGKVRAKAKSRSSRAGLQFPVGRVHRHLRKGNYADRVGAGAPVYLAAVLEYLTAEVLEAGNAARDNKKTRIIP	*J9WLZ9*	Antibacterial activity *A. hydrophila* 25 µM *V. cholera 25* µM *V. fluvialis 25* µM *S. aureus 25* µM	[88]
*Fragment H2A* *Sunetta scripta*	Sunettin 51 bp	12.25	MSGRGKGGKTKGKAKSRSSRAGLQFPVGRIHRLLRKGNYAERVGAGAPVYL	*G0YP03*	Apparent antibacterial activity	[56]
*Fragment H2A*, *Saccostrea cucullata* *molluscs*	Molluskin 25 bp	+6.25	SRSSRAGLQFPVGRIHRLLRKGNYA	*G0YP01*	Apparent antibacterial activity	[56]
*Fragment H2A* *Scylla paramamosain* *haemolymph*	Sphistin 38 bp	+11	MAGGKAGKDSGKAKAKAVSRSARAGLQFPVGRIHRHLK	*NR*	Antibacterial activity *M. lysodeikticus 1.5* µM *S.aureus 1.5* µM *B. subtilis 1.5* µM *C. glutamicum 1.5* µM *S.epidermidis 1.5* µM *S. flexneri 1.5* µM *A. hydrophila 1.5* µM *E. coli 1.5* µM Endotoxin activity binding LPS and LTA	[89]
H2B human Amniotic human fluid	Full length H2B 126 bp	+18.75	MPEPAKSAPAPKKGSKKAVTKAQKKDGKKRKRSRKESYSIYVYKVLKQVHPDTGISSKAMGIMNSFVNDIFERIAGEASRLAHYNKRSTITSREIQTAVRLLLPGELAKHAVSEGTKAVTKYTSSK	*Q16778*	Endotoxin activity binding LPS	[12]
*H2B* *Rhacophorus schlegelii* *Skin*	H2B 126 bp	+18.75	MPEPAKSAPAAKKGSKKAVSKVQKKDGKKRRKSRKESYAIYVYKVLKQVHPDTGISSKAMSIMNSFVNDIFERIAGEASRLAHYNKRSTITSREIQTAVRLLLPGELAKHAVSEGTKAVTKYTSAK	*Q75VN4*	Antibacterial activity *E. coli*	[42]
*H2B* *Litopenaeus vannamei* hemocytes	H2B 116 bp	+15.75	TSGKAAKKAGKAQKSITKGDKKKRKESYSIYIYKVLKQVHPDTGISSKAMSIMNSFVNDIFERIAAEASRLAHYNKRSTITSREIQTAVRLLLPGELAKHAVSEGTKAVTKYTSSK	*P83863*	Antibacterial activity *M. luteus 3* µM	[3]
Human H3.1	Full length H3 136 bp	+22	MARTKQTARKSTGGKAPRKQLATKAARKSAPATGGVKKPHRYRPGTVALREIRRYQKSTELLIRKLPFQRLVREIAQDFKTDLRFQSSAVMALQEACEAYLVGLFEDTNLCAIHAKRVTIMPKDIQLARRIRGERA	P68431	*Antimicrobial activity* *P. aeruginosa* 128 µg/mL *B. cereus* 32 µg/mL *C. albicans* 64 µg/m *E. aerogenes* 128 µg/mL *S. enterica* 128 µg/mL Endotoxin activity binding LPS and LTA	[66]
Human H3.1 fragment 1–34	H3DP1 34 bp	+10	ARTKQTARKSTGGKAPRKQLATKAARKSAPATGG		Endotoxin activity Binding LPS and LTA	[66]
Human H3.1 fragment 1–34	H3DP2 34 bp	+8.25	VKKPHRYRPGTVALREIRRYQKSTELLIRKLPFQ		*Antimicrobial activity* *E. coli 12*8 µg/mL *S. aureus* 128 µg/mL *C. albicans* 16 µg/mL Endotoxin activity Binding LPS and LTA	
Human H3.1 69–102 [81]	H3DP3 34 bp	−1	RLVREIAQDFKTDLRFQSSAVMALQEACEAYLVG		Non antimicrobial activity, neither endotoxin binding activity	
Human H3.1 103–135	H3DP4 33 bp	+3.25	LFEDTNLCAIHAKRVTIMPKDIQLARRIRGERA		Endotoxin activity Binding LPS and LTA	
Fragment of H3 *Designed*	DesHDPA2 20 bp	+5.25	HRYRPGTVALREIRRYQKST	*NR*	*Antimicrobial activity* *E. coli* 5.7 µM *S. marcescens* 1.0 µM *B. subtilis* 6.7 µM *S. aureus* 3.9 µM *E. faecalis* 1.1 µM	[34, 35]
Fragment of H4 *Designed*	DesHDAP3 22 bp	+6	KVLRDNIQGWTKPAIRRLARRG	*NR*	*Antimicrobial activity* *E. coli* 7.9 µM *S. marcescens* 1.0 µM *B. subtilis* 8.5µM *S. aureus* 3.7 µM *E. faecalis* 1.0 µM	

Histone derived-antimicrobial peptide (HDAP) reported for mammals, amphibian, fishes and several invertebrates

*LPS* Lipopolysaccharide, *LTA* Lipoteichoic acid, *NR* not reported

The first report of antimicrobial activity of histones and HDAPs was described in 1942 [18]. Then, Hirsch showed in 1958 that arginine-rich histones could kill both Gram-positive and Gram-negative bacteria [15]. As antimicrobial molecules, histones and HDAPs form part of the skin defense but are also isolated from other tissues, such as the stomach or intestine, reproductive tissue, and blood of different organisms. Additionally, histones are part of neutrophil extracellular traps (NETs), a protein complex in mammals that breaks down cells. NETs include neutrophil elastases, cathepsin G, and histones, and they help keep the balance between infection and immunity in the body [19]. NETs oversee disintegrating intracellular organelle membranes and disassembling foreign nuclear material.

The most studied histone is the core one, H2A, which is rich in basic amino acids, a characteristic that allows histone H2A to act as a precursor for several antimicrobial peptides. Structurally, HDAPs share all the essential traits of cationic antimicrobial peptides (AMPs); they are hydrophobic, cationic, and can form amphipathic alpha-helical structures [20–22].

With respect to their mechanism of action, AMPs commonly involve their ability to cause damage at the membrane level. Cationic AMPs can interact with microorganisms through electrostatic forces between the positive charge of their amino acids and the negative charge on the membrane surface. It has been suggested that the composition of the membrane surface is precisely what confers specificity to cationic AMPs. In this case, the sensitivity of cells, both prokaryotic and eukaryotic, is directly determined by the physicochemical properties of the lipids found in both types of membranes [23].

For example, in mammalian membranes, the most common lipids on the extracellular side of the bilayer are neutral phospholipids, such as phosphatidylcholine and sphingomyelin. The membrane of bacteria, on the other hand, is mostly made up of negatively charged lipids like cardiolipin and phosphatidylglycerol (PG) [24], as well as zwitterions like phosphatidylethanolamine (PE). Additionally, Gram-negative bacteria have lipopolysaccharides (LPS) in their outer membrane, and Gram-positive bacteria present acidic polysaccharides (teichoic acid and teichuronic acid) in the cell wall, which are also involved in the interaction with cationic AMPs. It has been demonstrated that the overall negative charge of bacterial membranes plays a significant role in the binding of AMPs to these microorganisms [24].

**Fig. 2:**
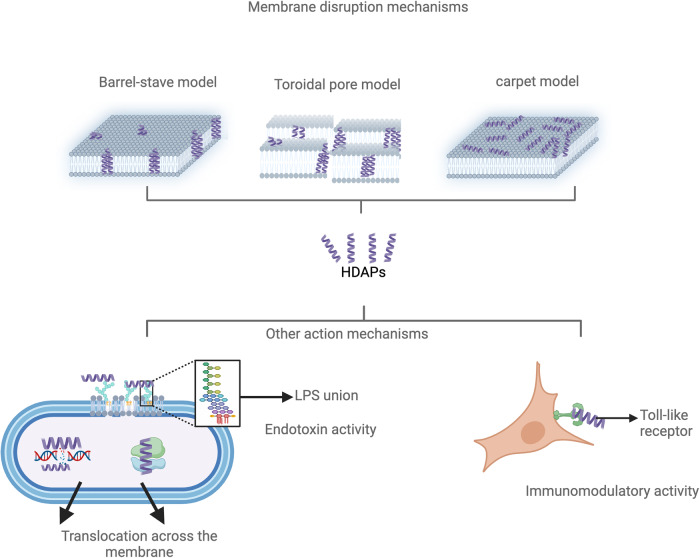
Reported mechanisms of action for antimicrobial peptides derived from histones. Among these are direct methods involving interaction and disruption of the membrane through pore formation and release of cellular content. The translocation mechanism targeting intracellular entities, as reported for buforin II, is also noteworthy. Similarly, neutralization and aggregation of toxins such as lipopolysaccharide (LPS) and lipoteichoic acid (TLA) are mentioned as anti-endotoxin mechanisms. Finally, the immunomodulatory functions are highlighted, often responsible for activating the inflammation cascade, which has been associated with adverse effects in various human tissues. “Created with BioRender.com”

The mechanisms by which cationic AMPs such as buforin II (BF2) can penetrate microbial cell membranes are not common to all peptides and depend on the molecular properties of both the peptides and the membrane lipids. AMPs can induce various types of damage to the membranes, including pore formation, phase separation, disruption of the lipid membrane, and micelle formation [25]. Several models have been put forward to explain how membrane disruption happens when AMPs interact with it. These models include the barrel-stave model, the toroidal pore model, and the carpet model [24] (Fig. 2).

## Barrel-stave model

This suggests that the peptides form a transmembrane pore by direct insertion into the lipid bilayer. In this model, the peptides initially bind to the membrane as monomers, which then aggregate and form a transmembrane pore similar to a bottomless barrel, with the helical peptides forming the walls of the pore. Additional recruitment of monomers increases the pore’s size, allowing the release of cytoplasmic content, resulting in osmotic imbalance and loss of membrane potential, ultimately leading to cell death. The peptides aggregate and insert into the bilayer in such a way that the hydrophobic regions of the peptides align with the lipid region of the membrane, leaving hydrophilic regions facing the central lumen. In this model, secondary structures, such as α-helices and β-sheets, are essential for pore formation [26].

## Toroidal pore model

A toroid is defined as a surface generated by a closed curve rotating around an axis contained in its plane without intersecting it. In the toroidal pore model, connected peptides aggregate and insert, inducing the lipid monolayer to curve through the pore, causing the polar regions of both membrane layers to merge. In contrast to the previous model, the polar heads of membrane lipids and the peptides inserted into it form a mixed channel. This type of mechanism has been found in peptides like Magainin (frog) and Melittin (bee) [27].

## Carpet model

In this model, peptides accumulate, covering the membrane surface like a carpet, affecting its architecture, and acting as a detergent. The peptides do not insert themselves into the membrane but associate with the outer face. When they reach a critical concentration, they disrupt the membrane’s continuity through their hydrophobic regions, breaking it down into micelles [28].

## HDAPs intracellular mode of action

AMPs had other mechanisms along with membrane penetration and pore formation, and HDAPs are representative of this. BF2 is the HDAP that has been studied the most. It is the same as a part of the N-terminal fragment histone subunit H2A. This peptide consists of 21 amino acids (TRSSRAGLQFPVGRVHRLLRK), comprising an N-terminal random coil region (residues 1–4), an extended helical region (residues 5–10), a hinge (proline residue 11), and a C-terminal regular a-helical region (residues 12–21) [29]. BF2, in contrast to most amphibian peptides, was isolated for the first time from the stomach tissue of *Bufo bufo gargarizans* but also from the skin secretions of *Sphaenorhynchus lacteus* [23]. Besides, it is a potent antibacterial that prevents bacterial growth without cell lysis, has a strong affinity for DNA and RNA, and modifies the gene expression profile in Gram-positive bacteria [23].To establish peptide translocation across the membranes without causing membrane permeabilization or lipid flip- flop, the Pro 11 appears to be a key structural factor for membrane penetration [30]. The role of Pro11 in the translocation of BF2 is crucial, as mutation of this residue abrogates the ability of the peptide to translocate across the membrane of *E. coli* [31]. Translocation capacity is a functional characteristic shared with cell-penetrating peptides (CPP) [30, 32].

Histones and HDAPs outside of cells act as natural barriers and have many antimicrobial properties that keep infections at bay. For example, they can break down bacterial cell membranes, enter cells and subsequently bind to bacterial DNA and/or RNA, attach to bacterial lipopolysaccharides (LPS) in the membrane, counteract the harmful effects of bacterial LPS, and trap pathogens as part of neutrophil extracellular traps (NETs). In accordance with the above, it is important to mention that all four core histones (H2A, H2B, H3, and H4), and H1, are demonstrated to be capable of crossing cell membranes as well as sometimes mediating the crossing of other small molecules covalently attached to them. This was demonstrated by Hariton-Gazal et al. [33] adding a mixture containing the five nucleosomal histones, H1, H2A, H2B, H3, and H4, as well as each of the last four individual histones, to intact HeLa and Colo-205 cultured cells resulted in cell penetration and nuclear import of these externally added histones. Furthermore, the authors demonstrated that the translocation was direct and without the endocytic pathway because various endocytosis inhibitors such as colchicine, nocodazole, cytochalasin D, brefeldin A, chloroquine, and nystatin did not have any effect on the penetration process.

On the other hand, it was found that not all HDAPs (DesHDAPs 1–3) previously described by Tsao et al. [34] use the same mechanism of action. The three peptides share sequence identity with a fragment of a histone subunit predicted to bind to DNA, firstly crossing the membrane without disrupted; DesHDAP1 is derived from H2A, DesHDAP2 from H3, and DesHDAP3 from H4. All the peptides also have several positive charges and one proline residue that breaks up a C-terminal helical region. Pavia et al. describe that DesHDAP2 and DesHDAP3 presented a strong cell penetrating mechanism, whereas DesHDAP1 did not present antibacterial activity by cell disrupting or cell-penetrating mechanisms [35].

The authors emphasize that the most studied of histones and their fragments is H2A, from which various fragments with antimicrobial activity have been discovered, such as parasin I, buforin II, and DesHDAP1. On the other hand, not as much is known about several fragments that can kill microbes through a mechanism that involves histones H3 and H4. However, Rosenbluh et al. [36] demonstrated the histone molecules’ ability to directly cross biological membranes via a non-endocytic pathway; they studied their ability to penetrate liposomes, mycoplasmas, and *E. coli* cells, as well as human erythrocyte.

On the other side, there is evidence of the antibacterial function of the histones intracellularly. It was discovered that histones can bind to lipid droplets—organelles in the cytosol that are primarily used to store energy—in various animal cells and tissues. It was also demonstrated that histones bound to lipid droplets can protect cells against bacteria without causing any of the harm normally associated with the presence of free histones. In vitro experiments with lipid droplets purified from *Drosophila* embryos showed that histones bound to lipid droplets and were meant to kill bacteria could be released in the presence of LPS, or lipoteichoic acid produced by them [37].

## Diversity of histone-derived peptides

In relation to their broad antimicrobial properties, these proteins or HDAPs displayed the inhibitory microbial activity in hemolycytes of shrimps (H2A, H2B, H4) [14]; in the liver, intestine, stomach, testes, skin, gills and epithelial mucosa of fish (H1) [38],H1-like protein [39], H2B and H1-like protein [40]; parasin -H2A N-terminal residue [41]; in the skin and stomach of amphibians (H2B) [42], buforin I-H2A N-terminal residue [29]; in the liver, ovary and oviduct of birds (H2A and H2B) [43], H1 and H2B [44]; in the reproductive track of cow (H2A, H2B, H3 and H4) [45]; in the sebocytes (H4) [46], placenta (H2A and H2B) [47]; intestinal mucosa (H1 and its fragments) [48, 49], and the amniotic fluid (H2B) of mammals [12].

The histone H1-like protein (fH1LP) from flounder (fish), which is consistently found in the testis and ovary, was tested for its antibacterial properties, and found to be very effective against several microorganisms. This included Gram-positive bacteria such as *Bacillus subtilis*, *Staphylococcus aureus*, and *Streptococcus iniae* (minimal effective concentrations [MECs] ranging from 2.8 to 30.0 μg ml^−1^), Gram-negative bacteria like *Aeromonas hydrophila*, *Escherichia coli D31*, *Vibrio parahaemolyticus* (MECs, 1.4–12.0 μg ml^−1^), and *Candida albicans* (MEC, 2.0 μg ml^−1^). In the case of healthy Atlantic salmon *(Salmo salar)* it is reported that histone H1 had antibacterial activity (*E. coli* 31 μg ml^−1^) in vitro from the liver, intestine, and stomach [38]. Similarly, Oncorhyncin II and III are derived from histone H1 *Oncorhynchus mykiss*.

Histone H2A is the most frequently reported precursor of AMPs due to its abundance of basic amino acid residues like Arg and Lys at its N-terminal and their remarkable antimicrobial properties. Similarly, in catfish (*Parasilurus asotus*), Parasin I is produced by the cleavage of the Ser19-Arg20 bond of histone H2A by Cathepsin D, which forms the skin mucus of the fish from the epithelial mucosal layer [41]. The release of these types of AMPs has been related to skin and mucosal injuries, and it is believed that they function either in the cytoplasm against intracellular pathogens or extracellularly by spreading to mucosal surfaces or tissue fluids after infection-induced cell lysis or apoptosis. This HDAP had potent antimicrobial activity against Gram-positive bacteria such as *Bacillus subtilis*, *Staphylococcus aureus*, *Streptococcus mutans* and *Pseudomonas putida* (1–2 μg ml^−1^); bacteria Gram-negative including *Escherichia coli*, *Salmonella typhimurium* and *Serratia sp*. (1–4 μg ml^−1^).

In marine fish, AMPs derived from fragments of histone H2A have been reported from several species, including hipposin from Atlantic halibut *Hippoglossus hippoglossus* (Gram-positive and Gram-negative bacteria 1.6 μg ml^−1^), rainbow trout *Oncorhynchus mykiss* [50], Himanturin from round whip ray *Himantura pastinacoides* [51], and teleostin from two marine teleost fishes [52], *Tachysurusjella* and *Cynoglossus semifasciatus*. Histone H2A derived AMPs have also been reported from marine invertebrates, including Pacific white shrimp *Litopenaeus vannamei* [53], scallop *Chlamys farreri* [54], abhisin from abalone *Haliotis discus* (Gram-positive and Gram-negative bacteria 250 μg ml^−1^) [55], Sunetin from marine Clam *Sunetta scripta* [51], and molluskin from a few other marine molluscs [56].

Moreover, buforins are H2A HDAPs, derived from buforin I (Gram-positive bacteria *Bacillus subtilis, Staphylococcus aureus* and *Streptococcus mutans*; Gram-negative *Escherichia coli*, *Pseudomonas putida* and *Salmonella typhimurium*) buforin II is a 21-amino acid AMP that displays more potent antimicrobial activity (2–4 μg ml^−1^) than does its parent peptide (4–8 μg ml^−1^). Cho et al. [21], show that histone-derived AMPs such as buforin I (39 aminoacids) are produced by proteolytic breakdown of histone in the toad stomach. Histone H2A is overproduced in the cells of toad mucosal cells, creating an excess of what is required for DNA packaging purposes [29]. These extra histone subunits are kept in cytoplasmic granules until they are released into the stomach lumen. There, pepsine C lisosymes break down the H2A subunit to make buforin I.

Interestingly, there is evidence that the presence of histone H2B quantified in the mid-trimester at 125 ng ml^−1^ in the amniotic fluid of pregnant women protects the fetus from LPS in consequence of bacterial infections. The amount of histone 2B in the amniotic fluid was inversely related to the amount of TNF-alpha produced by ex vivo-cultured amniocytes in response to LPS [12].

Additionally, HDAPS in combination (linkers H1 and H5; core H1, H2A, H2B, H3) extracted and purified from chicken erythrocytes (Avian blood contains nucleated erythrocytes with DNA packaged by histones). Histone H5, which possesses 38% similarity to histone H1, does not have a mammalian analog. Histone H5 is 190 amino acids in length, with a hydrophobic ratio of 28%, a total net charge of +61 and an isoelectric point of +12. The mixture of histones demonstrate the affinity of histones ranging from 5 μg to 7.5 μg for *S. aureus* [55], *E. faecalis* and *B. subtilis* lipoteichoic acids (LTAs) as well as for *E. coli*, *S. Typhimurium* and *P. aeruginosa* lipopolysaccharides (LPS). Also, pure erythrocyte-specific histone H5 was three to four times more effective at killing planktonic cells than the mixture of histones (H1, H2A, H2B, H3, H4, H5). It was also three to four times more effective at killing Gram-positive bacterial biofilms (16–31 μg ml^−1^) [57].

## Harnessing AMP synergies to combat antimicrobial resistance

Drug combination therapies are applied in many diseases; in the case of infectious diseases, it is supposed that this could be a strategy to eliminate resistant strains or even delay the evolution of drug resistance. In accordance with the above synergistic antimicrobial interactions, they could improve the efficacy of new and existing antimicrobial agents. An example of this is when LL-37 a membrane disruption peptide and H2A a cell penetrating peptide are paired together. These functions combine and produce irreparable damage by targeting two sites: the bacterial membrane and the cytoplasm of *E. coli* and *S. aureus* [58]. Likewise, the combination of Nisin (bacteriocin) and Buforin I presents synergy against *E. coli*, *S. epidermidis* and *B. subtillis* [59]. The naturally occurring co-localization of histones with antimicrobial peptides (AMPs) in immune cells suggests that histones may be part of a larger antimicrobial mechanism in vivo [60].

Additionally, synergism has been evaluated between peptides and traditional antibiotics against bacteria from clinically isolated multidrug-resistant strains [61]. In this study, different peptides, including buforin II, were evaluated both individually and in combination with antibiotics such as amoxicillin-clavulanate, ceftriaxone, and polymyxin, among others. It was found that buforin II exhibited potent activity against *E. coli* at 0.50 mg ml^−1^ and *P. aeruginosa* at 8 mg ml^−1^, as well as against MSSA strains at concentrations of 0.5–8 mg ml^−1^ and MRSA strains at concentrations of 1–8 mg ml^−1^. Conversely, the combination of buforin II with the mentioned antibiotics was not found to enhance their action but rather had a summative effect. However, in a more recent study [62], evaluated the susceptibilities (cross-resistance or collateral sensitivity) of antibiotic-resistant *E. coli* strains to antimicrobial peptides and found that antibiotic-resistant bacteria show a high frequency of collateral sensitivity to antimicrobial peptides, whereas cross-resistance is relatively rare. Describing all the synergistic interactions of these peptides in vitro can pose a challenge due to potential interactions with other immune components and physiological conditions that may not be fully considered in an in vitro assay [63].

## Role in infections and inflammatory processes

**Fig. 3:**
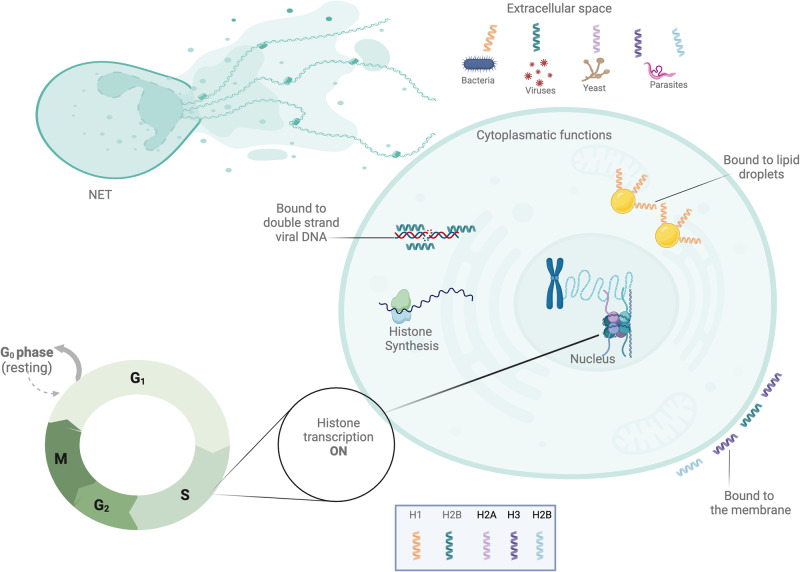
Described natural functions of histones. After being synthesized in the cytoplasm, histones undergo transportation to the nucleus, where they play a role in regulating DNA condensation and gene transcription. Alternatively, histones can serve extranuclear functions, either within the cytoplasm or in the extracellular space. Those transported from the cytoplasm may either remain bound to membranes or be released into the extracellular space, exhibiting a broad-spectrum antimicrobial activity against bacteria, viruses, parasites, and fungi. In the cytoplasm, histone H1 is associated with lipid droplets and is released upon stimulation with endotoxin or lipoteichoic acid. Additionally, histone H2B acts as a sensor for viral dsDNA. Nuclear histones ultimately contribute to neutrophil extracellular traps, playing a crucial role in neutrophil extracellular trap-mediated bacterial killing. “Created with BioRender.com”

The authors Allam et al. [64] and Hoeksema et al. [65], review that extracellular histones can be released into the extracellular space through various mechanisms, often associated with cellular stress, injury, or death. There are reported different forms to release histones to the extracellular space, principally by a necrotic process. These processes are represented in Fig. 3. We are mostly interested in Neutrophils extracellular traps that have to do with the immune system and getting rid of infections by breaking down full length histones.

Apart from acting as direct antimicrobial agents, extracellular histones play a role in host defense by being part of NETs. These traps consist of histones from nucleosomes and DNA released by neutrophils, forming external fibers on the cell surface. These fibers bind to both Gram-positive and Gram-negative bacteria, leading to the degradation of virulence factors and bacterial cell death [66]. The formation of NETs leads to the death of neutrophils, which is a unique way for cells to die that is different from both necrosis and apoptosis. Approximately seventy percent of the proteins forming these NETs are core histones (H2A, H2B, H3, and H4), and they are thought to contribute to biological defense through their antimicrobial properties as components of NETs.

Another well-known property of histones is that they can interact with endothelial cells, which can cause calcium to flow into the cell and cause cell death. Elevated histone levels (50 µg ml^−1^ free histones) in the serum have been observed in patients experiencing severe trauma, pancreatitis, or sepsis [67]. Histones can trigger inflammasome activation by specifically binding to TLR2 and TLR4, which are the primary receptors for extracellular histones. Subsequent activation contributes to endothelial dysfunction, organ failure, and death during sepsis. The signaling pathways of extracellular histones through TLRs present potential novel therapeutic targets for a range of inflammatory and toxin-mediated diseases [65].

Despite these adverse reactions to the presence of free histones, primarily released during necrotic processes, it is important to note that histones with antibacterial activity have been observed to reduce their potential to elicit immune system adverse reactions through acetylation or association with cytoplasmic lipids, thereby diminishing their alarming potential damage reported in endothelial cells and activation of the coagulation cascade.

Based on what has been said so far and what has been found in vitro, it seems that these AMPs have other biological effects. These effects include the inhibition of botulinum neurotoxins [68] and the coagulation initiated by tissue factors [69]. Notably, several AMPs exhibit anti-endotoxin activity, which results from the peptide’s binding to bacterial lipopolysaccharide (LPS) and lipoteichoic acid. This activity prevents the development of sepsis and septic shock associated with pathogenic Gram-negative and Gram-positive bacteria [15, 70, 71]. Similarly, buforin II has the ability to prevent lethal endotoxemia in a rat model of peritonitis [72–74]. In this model, administering a single dose of buforin II reduces intra-abdominal bacterial concentration and mortality. Additionally, the concentrations of LPS and the LPS-induced host cytokine TNF-α, which mediates sepsis, are dramatically decreased in the blood of septic rats treated with buforin II. These rats had intra-abdominal sepsis induced through cecal ligation and a single puncture.

## Future perspectives and therapeutic applications

The objective of this section is to illustrate that notwithstanding the potential of AMPs, including those derived from histones, and the imperative for novel antibiotics considering the resistance crisis to conventional antibiotics, the number of these molecules currently in clinical application is restricted. In accordance with this, we will review the possible reasons for this and potential solutions. Currently, nisin, gramicidin, polymyxins, daptomycin, and melittin are employed clinically as antibiotic alternatives due to their potent antimicrobial properties. Presently, no histone-derived peptides (HDPs) are undergoing clinical trials or in active use. For instance, nisin targets Gram-positive bacteria and is administered orally. Gramicidins and polymyxins, which are specifically formulated for topical application, are employed to treat Gram-negative bacteria and infected wounds, respectively [75].

Despite the resolution of concerns related to pharmacokinetics and safety, several AMPs encountered obstacles throughout Phase III clinical trials. These setbacks were primarily caused by the lack of demonstrated efficacy or the inability to outperform conventional treatments. A case in point is the AMP Neuprex® (rBPI21), a recombinant α-helical peptide derived from the N-terminus of BPI, encompassing the initial 193 amino acids [76]. In parallel to Neuprex®, at least five other AMPs that completed advanced clinical trials also failed to exhibit clear efficacy (e.g., iseganan and XOMA-629) or superiority over conventional treatments (e.g., surotomycin, pexiganan, and omiganan) [77]. Moreover, the drawbacks in AMPs clinical trials may arise from stability issues, inappropriate drug administration or unknown interactions between the peptide and the standard treatment. As well as high development and production costs ($50–400 per gram of amino acid for AMPs by solid phase synthesis compared with $0.80 per gram of aminoglicoside production), cytotoxic issues, reduced activity in clinically relevant environments (half-life and stability), and the emergence of bacterial resistance, despite the initial claims that they may not induce resistance [78].

Diverse strategies have been suggested to overcome the obstacles associated with the research, development, and clinical application of AMPs. Alternative peptide synthesis methods and the production of ultra-short and/or truncated AMPs are being considered to reduce production expenses. Conversely, solution phase synthesis or chemoenzymatic methods could be used to produce small AMPs and for larger AMPs, the considered option is recombinant production.

As a solution, have been documented to enhance bioavailability, stability, efficacy, and specificity while decreasing cytotoxicity, modification such as PEGylation, lipidation.

Finally, recapitulating the HDPs and their capacity to both interact with bacterial membranes and cell trespassing on intracellular targets (CPPs) means that these molecules act as candidates to new antimicrobial therapies, along with nanoplatform systems to deliver them. Another important advantage of CCPs is their ability to transport cargo to intracellular compartments of the cell. CPPs have also been conjugated to cargos with many different sizes and efficiently transport both in vitro and in vivo peptides, proteins, antibodies, nucleic acids, fluorochromes, nanoparticles, lipid-based formulations, viruses, contrast agents for magnetic resonance imaging, and drugs. In this context, the buforin II represents a histone-derived peptide that acts as a cell-penetrating peptide, translocating spontaneously across membranes. Recent studies have determined the potential of buforin II-magnetite conjugates as cell-penetrating vehicles for the targeted delivery of pharmacological agents [79].
